# Microbiota and Probiotics in Health and HIV Infection

**DOI:** 10.3390/nu9060615

**Published:** 2017-06-16

**Authors:** Chiara D’Angelo, Marcella Reale, Erica Costantini

**Affiliations:** Unit of Immunodiagnostic and Molecular Pathology, Department of Medical, Oral and Biotechnological Sciences, University “G. d’Annunzio” Chieti-Pescara, 66100 Chieti, Italy; chiara.dangelo@unich.it (C.D.); erica.costantini@unich.it (E.C.)

**Keywords:** microbiome, probiotics, dietary supplements, nutrition, HIV, inflammation

## Abstract

Microbiota play a key role in various body functions, as well as in physiological, metabolic, and immunological processes, through different mechanisms such as the regulation of the development and/or functions of different types of immune cells in the intestines. Evidence indicates that alteration in the gut microbiota can influence infectious and non-infectious diseases. Bacteria that reside on the mucosal surface or within the mucus layer interact with the host immune system, thus, a healthy gut microbiota is essential for the development of mucosal immunity. In patients with human immunodeficiency virus (HIV), including those who control their disease with antiretroviral drugs (ART), the gut microbiome is very different than the microbiome of those not infected with HIV. Recent data suggests that, for these patients, dysbiosis may lead to a breakdown in the gut’s immunologic activity, causing systemic bacteria diffusion and inflammation. Since in HIV-infected patients in this state, including those in ART therapy, the treatment of gastrointestinal tract disorders is frustrating, many studies are in progress to investigate the ability of probiotics to modulate epithelial barrier functions, microbiota composition, and microbial translocation. This mini-review analyzed the use of probiotics to prevent and attenuate several gastrointestinal manifestations and to improve gut-associated lymphoid tissue (GALT) immunity in HIV infection.

## 1. Introduction

Over the past 20 years, the increasing interest in the health effects of probiotic consumption has erupted in studies both in food and pharmaceutical companies, and studies have been conducted to understand the effects of probiotics on the regulation of the immune response and potential applications for disease prevention. Probiotic benefits are not a recent discovery: they were already present a long time ago in traditional foods, such as cheese, yogurt, milk, and salty fishes, and used for nutritional purposes. Subsequently, people noted the beneficial health effects of eating fermented foods. 

Over the years, probiotics have been described as “organisms and substances which contribute to intestinal microbial balance” [1,2,3]. For the Food and Agriculture Organization/World Health Organization (FAO/WHO), the term probiotic is defined as “live microorganisms which, when administered in adequate amounts, confer a health benefit on the host”.

Improving health could be a useful strategy for protecting us from several illnesses, and probiotics are able to enrich our digestive system with good microbes that are able to neutralize the harmful ones and restore the balance between bacteria such as lactobacilli, streptococci, clostridia, coliform, and bacteroides. Thus, probiotics may confer a health benefit on the host by the modulation of the immune system [4,5], limiting pathogen colonization [6,7], and controlling inflammatory gut disorders [8] and metabolic disorders [9]. Probiotics are also helpful during antibiotic administration—reducing antibiotic-associated diarrhea—and in restoring normal gut permeability, mechanical integrity, and homeostasis [10].

Some effects attributed to probiotics have been proved by clinical trials, and the effectiveness of probiotics has been demonstrated in disorders, such as inflammatory bowel diseases (IBD), diarrhea, allergies, and the prevention of upper respiratory tract infections [11,12,13], and also in unbalanced conditions of intestinal flora induced by stress, lifestyle, host genetics, inadequate food, and exposure to environmental toxins [14,15,16].

Many studies have demonstrated that the human immunodeficiency virus (HIV) has harmful effects on the human immune system, mainly on the cluster differentiation (CD4)^+^ T-cells, and that HIV infection is characterized by gut microbiota dysbiosis, an altered intestinal barrier, and systemic inflammation [17,18,19,20]. The mucosal immune system can be modulated by gut-resident bacteria, and alteration of the mucosal innate immune system can result in the outgrowth of a dysbiotic pro-inflammatory group accountable for chronic inflammation in the mucosa and the periphery [21,22]. HIV infection significantly alters total microbial colonization as well as the microbiota composition in the oral cavity, and decreased CD4 cell counts have been associated with the presence of oral lesions [23].

Progressive HIV infection is characterized by the dysregulation of intestinal immunity that may also persist during highly-active antiretroviral therapy, and the extent of the gut and oral microbiota dysbiosis correlates with markers of disease progression [24,25].

Thus, interventions in HIV-positive patients are necessary to restore the integrity of the immune system of gut-associated lymphoid tissue (GALT), and the use of probiotics may recover gut barrier functions, remodel the microbiome, and aid to decrease bacterial translocation and pro-inflammatory cytokine production, thereby improving immune functions in HIV-infected subjects, including during short-term antiretroviral therapy (ART) [26,27,28].

Mechanisms by which probiotics may exert their effects are strain-related and include the host’s microbiota modulation, improvement of mucosal barrier functions, and modulation of the immune system [29,30]. As all the implicated mechanisms are not completely known, probiotic clinical use needs to be related to probiotic strain and dosage, in order to identify their efficacy under specific conditions [31]. Studies have been conducted, and others are in progress, with the aim of understanding probiotic-specific mechanisms and selecting probiotic strains in relation to the target patient’s specific pathogenic and clinical defects [32,33].

## 2. The Intestinal Microbiota Functions

The total human body surface, lung, oral and vaginal mucosa, and the gastrointestinal (GI) tract host over 10^14^ microorganisms—starting from birth—which form the microbiota. About 99% of the microbiota is present in the GI, achieving a configuration during human evolution, and has a major impact on the gastrointestinal tract and mucosal immune functions, and significantly affects the health of their host. For this reason, the gastrointestinal microbiome is the best-investigated microbiome and serves as a model for understanding host–microbiota interactions and disease. The development of next-generation DNA sequencing platforms has clarified the composition of the intestinal microbiota, that is, a complex microbial ecosystem. Under healthy conditions, it includes different species of bacteria, each of which contains many functionally different strains with significant genetic diversity. The majority of strains are strictly anaerobes, even if facultative anaerobes and aerobes are present. Some bacterial strains are prevalent: fermenting bacteria (such as *Lactobacillus* and *Bifidobacteria*) represent 80% of the gut microbiota, while the remaining 20% includes *Escherichia*, *Bacteroides*, *Eubacteria*, and *Clostridium*. Lactic acid bacteria (LAB) are considered a major group of probiotic bacteria and have been isolated directly from humans. To date, different bacterial genera are known, including *Bifidobacterium* and *Lactobacillus*: they survive stomach acid pH and intestinal bile salts, reach sites of action, and their ingestion does not cause any risk for the host. It is known that a healthy gut flora is largely responsible of the overall health of the host, while gut microbiota alteration is associated with several human diseases, such as bowel diseases, metabolic and allergic diseases, or neurodevelopmental illnesses [34,35,36]. Thus, researchers are beginning to consider intestinal microbiota as another organ of the human body with different functions, such as maintenance of the epithelial barrier, inhibition of pathogen adhesion to intestinal surfaces, and modulation of the immune system [37].

### 2.1. Function and Preservation of the Intestinal Barrier

The GI mucosal surface is the largest area of the body in contact with the external environment; it plays a key role in blocking the access of potentially harmful substances. The epithelium and the mucus layer, lining the gut, represent the host’s first line of defense and the essential mechanical barrier that avoids contact between the internal and the external environments by blocking the passage of antigens, toxins, and microbial products, thus acting as a component of innate immunity [38]. 

The intestinal barrier is equipped with several levels of defense mechanisms to limit luminal antigen translocation. In a normal gut, the epithelial barrier consists of a layer of enterocyte tight junctions, anchoring junctions, and desmosomes—which hinder microbe passage—goblet cells producing mucus, and Paneth cells. Intestinal epithelial cells (IECs) can sense and respond to microbial stimuli, support barrier functions, and participate in immune responses [39,40]. The function of the epithelial barrier depends on junctional complexes formed by transmembrane proteins, such as claudins that form paracellular channels for small cations and water. Yuan et al. [41] showed the changes in expression and distribution of claudin proteins, which are essential for the formation and the integrity of tight junctions, which regulate the flow of water ions and small molecules, and their relationships with barrier dysfunction.

Paneth cells may limit bacterial penetration through pattern recognition receptors (PRR) and secretion of mucins and antimicrobial proteins (AMPs), establishing a physical and biochemical barrier to microbial penetration and underlying immune cells [42]. Intestinal epithelial cells produce immunoregulatory signals for tolerizing immune cells, limiting steady-state inflammation, and directing innate and adaptive immune cell responses against pathogens and commensal bacteria. Specialized epithelial cells, called M-cells, mediate the constant sampling of luminal antigens, and both microorganisms and macromolecules can gain entry through the M-cells [43]. 

Commensal bacteria induce cytokine production by IECs via PRR signaling, promoting the development of dendritic cells (DC) and macrophages with tolerogenic properties [44,45]. Commensal microorganisms may regulate barrier functions, controlling mucus production by goblet cells [46] or the expression of AMPs. Intestinal epithelial cells secrete Immunoglobulin (Ig)A in the lamina propria and express microbial recognition receptors, such as Toll-like receptors (TLR), that can recognize both antigens derived from the microbiota or invading pathogens. Under homeostatic conditions, IECs are unresponsive to TLR stimuli, while increased TLR expression was observed under inflammatory conditions. TLRs act as a link between microbiota alterations and immune homeostasis [47]. TLRs promote epithelial cell proliferation, secretion of IgA into the gut lumen, the expression of antimicrobial peptides [48], and play a role in intestinal barrier homeostasis [49]. The expression of tight junction proteins was modulated by TLR activation [50], and during inflammatory disorders epithelial tight junctions are impaired and result in increased bacterial translocation into the lamina propria, supporting the inflammatory response.

Many factors can alter the intestinal permeability and GI infections may be responsible for altered nutrient absorption, depleted levels of micronutrients, and waste secretion. As a consequence of microbe activity and the release of soluble peptides or toxins, there are alterations in enterocyte components and their metabolism, leading to a breakdown of the epithelial barrier and to microbial translocation in the gut [51]. Moreover, lifestyle and dietetic factors, including alcohol and energy-dense foods, can increase intestinal permeability [16]. The resulting increased permeability does facilitate chronic intestinal inflammation, strictly connected to the immune system, as observed in the existing association between inflammation and barrier dysfunction in several GI diseases. The proper defense activity of the epithelial barrier is supported by the microbiota, which influences cell metabolism and proliferation, maintenance and repair of barrier integrity, nutrient acquisition and energy regulation, inflammatory response, and angiogenesis [52,53,54].

The intake of probiotics can reduce the risk of diseases associated with intestinal barrier dysfunction. The mechanisms by which probiotics can influence the barrier function are also an area of interest, although many studies have shown that probiotics increase the barrier function by increasing mucus, antimicrobial peptides, and secretory IgA production, as well as increasing competitive adherence for pathogens, and the tight junctions (TJ) integrity of epithelial cells [55,56,57]. It is known that certain lactobacilli adhere to mucosal surfaces, inhibiting the attachment of pathogenic bacteria and enhancing the secretion of mucin. 

### 2.2. Resistance to Pathogenic Colonization

One of the major functions of the intestinal microbiota is the protection of the host from colonization and overgrowth of ingested invading bacteria, a phenomenon known as resistance to colonization [41]. Endogenous microbial populations act via several mechanisms, including the modification of the pH in the environment and ecological niches, the release of antimicrobial substances, and the direct competition for the adhesion sites on the epithelium and for nutritive substrates.

After ingestion, pathogens penetrate the highly-colonized mucus layer, where they compete with the resident microbiota for adhesion to the intestinal epithelial cell receptors. In healthy subjects, the direct competition for nutrients limits the possibilities for exogenous pathogenic microbes to colonize and replicate within the gut lumen and invading deeper tissues [58]. Additionally, the production of pathogen growth inhibitors or the resistance to colonization, due to the induction of immune responses and to metabolic products of beneficial bacterial, makes the host resistant to pathogenic infections. In addition, in the GI tract, the microbiota affects biosynthesis and the availability of neurotransmitters that modulate peristalsis, the flow of blood, and the secretion of ions [35,36].

Traditional probiotic approaches to maintain colonization resistance are designed to modulate the competition for nutritious substrates and adhesion sites, as well as the prevention of microorganism translocation and stimulation of the immune system. 

### 2.3. Development and Stimulation of GALT 

The presence of the microbiota is crucial for the normal development of GALT. Already from birth, the presence of intestinal microorganisms stimulates GALT to recognize the conserved microbial structures, ensuring an appropriate immune activity. GALT composition is modified immediately after microbial colonization of the GI tract, with a number of intraepithelial lymphocytes and immunoglobulin-producing cells in follicles and in the lamina propria. Bacterial antigen detection is performed by the resident cells of the innate and adaptive immune system. Signals from bacteria can be transmitted to macrophages, dendritic cells, and lymphocytes through molecules expressed on the epithelial cell surface, such as molecules of the major histocompatibility complex I and II, Toll-like receptors, and nucleotide oligomerization domain (NOD)-like receptors or nucleotide-binding domain leucine-rich repeat-containing (NLRs) proteins [59]. Antigen-presenting cells (APCs) provide processed antigens to naïve lymphocytes within distinct T- and B-cell zones. 

Mucosal effector sites consist of T lymphocytes, primarily CD8^+^, located in the epithelium and in the lamina propria, and CD4^+^ T-cells and plasma cells that heavily populate the large and small intestines, beneath the lamina propria. 

The CD4^+^ T lymphocytes can differentiate into T helper (Th)1, Th2, Th17, and regulatory T (Treg) cells. CD4^+^ Th17 cells share differentiation pathways and a reciprocal relationship with antigen-induced cells and CD4^+^ Treg cells, which are both able to maintain the balance between inflammation and tolerance. Th17 cells, characterized by the production of cytokines interleukin (IL)-17A, IL-17F, and IL-22, which have their receptors on epithelial cells [60,61], are specialized in maintaining mucosal integrity, stimulating the proliferation of epithelial cells, producing tight junction proteins (claudins), and modulating a robust antimicrobial inflammatory response by neutrophil and macrophage recruitment via chemokine, antimicrobial defensins, and mucin production [62,63,64,65].

Treg cells, maintaining immune homeostasis, have anti-inflammatory activity and prevent autoimmunity, inducing tolerance against self-antigens. Without an inflammatory stimulus, commensal microorganisms induce tolerogenic maturation of DCs, leading to the induction of various types of Treg or hypo-responsive T-cells [66]. 

Humoral immune response represents the main mechanism of protection given by GALT, mediated also by B cells secreting IgA, of which the intestinal DCs are potent inducers. It has anti-pathogenic effects and prevents commensal bacteria penetration in the host [67]. 

Epithelial cells, APCs, and lymphocytes can secrete cytokines, chemokines, and other factors that can be tuned to promote tolerance, inflammation, or specific immunity.

The dualistic effect that the microbiota exerts on GALT consists in maintaining tolerance and preventing inflammation through β-defensins and IgA production in the epithelium, whose integrity is enhanced through TLR signaling and Treg induction [45]. The equilibrium between microbiota, immune response, and tolerance mechanisms is important for a healthy intestine, and an aberrant colonization may drive mucosal inflammation, which plays a pivotal function in the development of feeding intolerance. The constant interplay between the microbiota, the intestinal barrier, and the mucosal immune system ensures the balance between permissive or tolerogenic responses to pathogens or food antigens [68]. 

Probiotics may induce a tolerogenic situation by modulating anti-inflammatory/regulatory cytokines, such as IL-10 and transforming growth factor (TGF)-β, and DC functionality. The supplementation with specific probiotics can promote the restoration of the intestinal CD4^+^ T-cell population in many immunological diseases, while the anti-inflammatory effects of probiotics in Th17-related diseases might be a consequence of the downregulation of pro-inflammatory IL-17 production [38]. 

## 3. Bowel Conditions in People Living with HIV 

The GI tract is a major site of HIV replication, and its disorders are among the most frequent complaints in patients with HIV infection. Patients with HIV infection are susceptible to gastric hypoacidity, which may be responsible for a greater risk of opportunistic infection. Additionally, delayed gastric emptying may contribute to the increased bacterial colonization of the upper digestive tract, playing a key role in chronic diarrhea and weight loss, and dysphagia and odynophagia, in which nausea, vomiting, and abdominal pain are the most frequent symptoms [69,70]. HIV infection has an unfavorable effect on the interaction between the commensal microbiota and the immune system, with progressive immune decline associated with inefficient epithelial repair and enhanced epithelial permeability responsible for GI disorders [69]. In people with HIV infection or acquired immune deficiency syndrome (AIDS), the wall of the small intestine is impaired, the crypts are enlarged, and the atrophy of the microvilli decreases their surface area. These modifications are responsible for malabsorption, digestive discomfort, or decreased intake of nutrients. 

HIV infection causes a breakdown of the GI barrier, alters the homeostatic balance between GI bacteria and gut immunity, and induces a compositional shift of gut microbiota [71] with the enrichment of either pro-inflammatory or potentially pathogenic bacterial populations [72], such as *Pseudomonas aeruginosa* and *Candida albicans*, and the reduction of *Bifidobacteria* levels and *Lactobacillus* species. These bacterial populations are associated with damage and loss of mucosal barrier functions [73,74] that are correlated with immune status [25,75]. In HIV infection, the increased translocation of microbes and bacterial products from the intestinal tract may induce a systemic immune activation, which causes further damage to the gut barrier function, augmenting bacterial translocation and subsequently increasing systemic inflammation and, in turn, HIV progression [76,77].

Throughout the initial stage of HIV infection, the immune system is unprepared for the attack of the virus, which therefore reproduces at very high levels in the lamina propria, spreading throughout the body. HIV causes a disruption of gut microbiota and 50% of lamina propria CD4 cells are depleted in early and acute HIV infection [19], as these cells may be more susceptible to HIV infection due to high levels of activation and expression of C-C chemokine receptor (CCR)5 receptors [78], in particular the CD4 cells that produce IL-17 and IL-22. The mechanism of this depletion is likely cell death of productively infected cells via apoptosis as well as of bystander cells via pyroptosis and the direct killing of infected cells by natural killer (NK) cells or cytotoxic T-cells [79,80]. The combination of these mechanisms may contribute to CD4^+^ T-cells loss, mucosal barrier damage, and chronic systemic inflammation. The consequences of reduced CD4 cells is the failure of gut mucosal barrier to protect against invading pathogens as well as the loss of cytokines necessary to support normal barrier function. Usually, with <100 CD4^+^ T-cells/mL, opportunistic infections of pathogenic bacteria and/or fungi drive GI dysfunctions, and HIV-1 directly drives mucosal inflammation, causing HIV-related enteropathies [81]. Poor CD4 recovery is linked to microbial translocation, and in HIV-infected persons with poor CD4 recovery, intestinal barrier dysfunction and mortality has been linked to elevated plasma kynurenine/tryptophan ratio [82]. 

The existence of HIV-specific IL-17-producing CD4^+^ T-cells, named Th17, have been reported [83,84], but it was not completely determined whether Th17 cells have direct anti-viral functions during HIV infection. Th17 and Th22 cells could play a role in amplifying the innate responses to HIV infection by enhancing the production of IL-22, a critical cytokine for epithelial barrier maintenance, which enhances epithelial regeneration inducing stem cell–mediated epithelial cell proliferation [85], and the expression of anti-microbial peptides [65].

**Figure 1:**
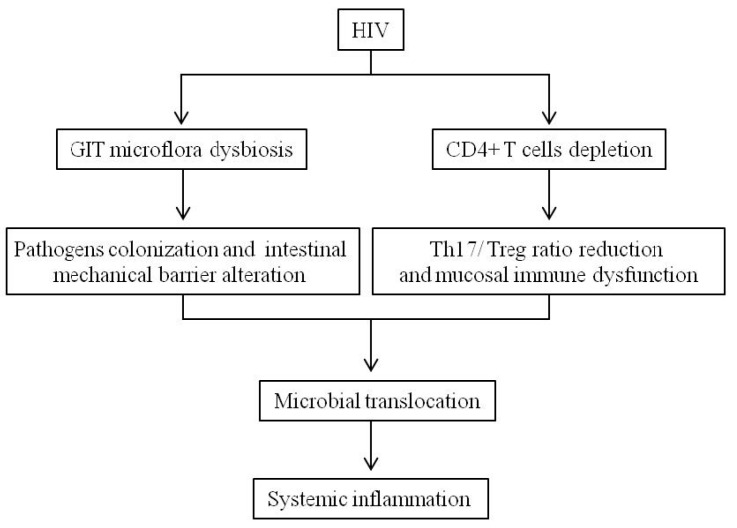
Gastrointestinal tract dysfunctions in HIV-infected patients. HIV: human immunodeficiency virus; GIT: gastro intestinal tract; Treg: T regulatory cells.

During HIV infection, high levels of viremia are associated with an important Th17 reduction in the gut; the loss of mucosal Th17 cells may be related to a decrease in mucosal restoration and an increase of microbial translocation from the gut lumen to the systemic circulation and immune hyperactivation, contributing to the exacerbation of the infection and to opportunistic infections [86,87,88]. The loss of Th17 cells was accompanied by a concomitant rise of Treg cells, resulting in an imbalanced Th17/Treg ratio during HIV progression [89,90,91]. A low Th17/Treg ratio in HIV-infected individuals correlates with microbial translocation and with a higher frequency of activated CD8^+^ T-cells, which is one of the strongest predictors of mortality. Treg cells may have both a beneficial and a detrimental role; the first is by limiting immune activation, while the second is based on the ability of Treg cells to suppress virus-specific immune responses. Thus, the role of Treg cells in regulating T-cell activation in HIV infection is still debated [92] (Figure 1). 

HIV infection is associated with an inflammatory state, as evidenced by high levels of Tumor necrosis Factor (TNF) and Tumor necrosis Factor Receptors (TNFRs) 1 and 2, IL-6, and Interferon (IFN)α [93] that may also lead to tight junction destruction. These changes may lead to impaired barrier function [94] and intestinal permeability with an increase of markers for microbial translocation/monocyte activation, such as lipopolysaccharide (LPS) and soluble CD14 into the plasma. Brenchley et al. [76] reported that plasma LPS levels and bacterial ribosomal DNA were elevated in patients with HIV infection compared with healthy controls, and circulating microbial products have been appointed as a possible cause of HIV-related systemic immune activation, HIV progression promotion, and suboptimal response to therapy and co-morbidity. Chronic TLR activation in HIV disease, through recognition of translocated bacterial products and/or viral products, can cause the dysregulation of immune responses. 

## 4. Probiotics as a New Therapeutic Approach That Might Improve Life in HIV-Positive Subjects 

Although ART and other pharmacological therapies are life-saving in HIV-positive subjects, due to the suppression of plasma viremia, the number of mucosal CD4 cells does not always fully recover, and microbial translocation is still not under full control and remains associated with systemic immune activation and inflammation, characterized by elevated pro-inflammatory cytokine levels, as well as T and B cell activation and tight junction dysfunction between the epithelial cells of the mucosal barrier.

Epithelial barrier dysfunction, measured by peripheral blood levels of intestinal fatty acid-binding protein and zonulin-1, predicted mortality in HIV infection, even after adjustment for CD4 count [95]. 

Several HIV-affected patients may be effectively managed by controlling the HIV infection with high-efficacy and improved ART, while other HIV-positive patients have many side effects, such as diarrhea and other GI symptoms associated with a worse quality of life, leading to a discontinuation of treatment and the requirement of more complex approaches [96,97,98,99]. 

The hypothesis that probiotic administration protects the gut surface and can delay the progression of HIV infection to AIDS was proposed some years ago. The use of probiotics may be inexpensive and potentially useful to reduce HIV-related morbidity and mortality [100].

**Figure 2:**
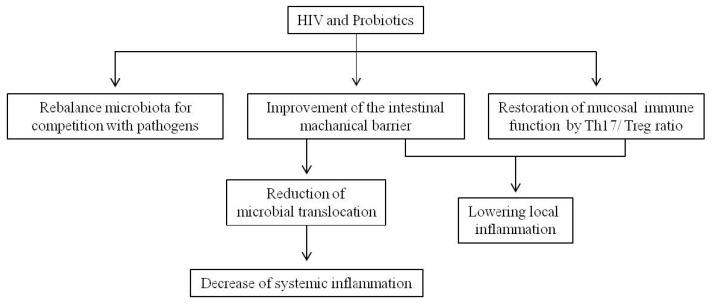
Probiotics use and beneficial effects in the gastrointestinal tract of HIV-1-infected patients.

There are many possible mechanisms by which probiotics may interfere with HIV (Figure 2). Probiotics can compete for nutrients and epithelial and mucosal adherence, inhibit epithelial invasion, counteract the inflammatory process by stabilizing and strengthening the gut microbiota responsible for the intestinal barrier integrity, prevent microbial translocation, lower mucosal and systemic inflammation, stimulate production of antimicrobial substances [101,102,103], and promote intestinal immunoglobulin A responses to improve the immunological barrier function [104,105,106,107]. The effectiveness of diet supplementation with different probiotic strains has been shown in people with HIV and has especially been shown as an additional strategy in patients on ART, in order to improve antioxidant defenses and aid in the reconstitution of the immune function. 

Gut reconditioning through probiotic administration could be protective of the gut surface and delay the progression to AIDS [108]. Probiotics, by altering intestinal flora, may induce epithelial healing, and by preventing the decline in CD4^+^ cell counts may lower the risk of virus transmission and reduce hospitalization for co-infections. ART-treated patients who fail to have an immunologic response (CD4 < 200) have lower levels of lactobacilli, elevated levels of LPS and sCD14, and increased inflammatory markers, such as IL-6 and sCD14 [109,110]. In 2010, Irvine et al. ran an observational retrospective study to assess the effect of a *Lactobacillus rhamnosus* Fiti yogurt on CD4^+^ cell counts in HIV subjects; the study showed an increased CD4^+^ cell average count over a period of three years in yogurt consumers [111]. Gori et al. reported that, in Highly Active Anti-Retroviral Therapy (HAART)-naïve HIV-infected patients, dietary supplementation with a prebiotic mixture results in the improvement of gut microbiota composition, the reduction of sCD14, CD4^+^ T-cell activation (CD25), and improved NK cell activity [28]. The study of Kim et al. evaluated the ability of probiotics, provided during combined antiretroviral therapy (cART), to reduce inflammation and improve gut immune health in HIV-positive treatment-naïve individuals (PROOV IT I) and in individuals with suboptimal CD4 recovery on cART (PROOV IT II) [108]. 

A combination of probiotic bacteria upregulates Treg cell activation and suppresses pro-inflammatory immune responses in models of autoimmunity, including IBD, thus providing a rationale for the use of probiotics in HIV infection.

In addition to the ability of probiotics to improve barrier function and intestinal homeostasis, specific probiotic strains may be able to revert the HIV-induced Th-2 polarization [112]. The study carried out by d’Ettorre et al. in 2015, where HIV-infected patients on ART were supplemented with probiotics, showed that inflammation and markers of microbial translocation were significantly reduced [101]. In HIV-infected subjects, diet supplementation for four weeks with *Lactobacillus casei* Shirota were virologically, bacteriologically, and immunologically beneficial, leading to increased levels of CD56^+^ cells and to a reduction of inflammatory status with significantly increased IL-23 serum levels. In addition, probiotic supplementation could be useful in the reduction of risk factors for cardiovascular diseases, such as hypercholesterolemia, as well as in the improvement of quality of life by improving the nutritional status, alleviating GI manifestations, and stimulating mucosal immune function [103].

Bacterial vaginosis may increase the risk of transmission or acquisition of HIV, increasing proinflammatory cytokines and disrupting the mucosal barrier function [113], and probiotic intervention may be prophylactic for bacterial vaginosis [114]. 

In HIV-affected patients, a periodontal disease, an extensive dysbiosis in the oral microbiome is a comorbidity that could act as a font of chronic inflammation, or a risk of various systemic diseases such as diabetes, hyperlipedemia, chronic kidney diseases. Recently, various studies have reported the lactic acid inhibition of oral bacteria, suggesting a promising role in combating periodontal diseases. Thus, in HIV-affected patients, probiotics may be a low-cost and accessible treatment approach to periodontal diseases that confer benefits upon host well-being, improving the quality of life [115,116,117,118]. 

## 5. Conclusions

The helpful effects of probiotics to maintain our body in health are well-known, and several clinical and in vitro studies have shown a large field of application for probiotic supplementation related to benefits that occur in infections and diseases [119,120,121]. Probiotics reduce gastrointestinal discomfort and reinforce the various lines of gut defense: immune exclusion, immune elimination, and immune regulation. Probiotics also stimulate non-specific host resistance to microbial pathogens and thereby aid in their eradication, maintaining a ‘healthy’ microbiota [122].

The intestinal microbiome has been proposed as a novel therapeutic target for reducing chronic inflammation [78,123], and probiotics have been proposed to improve the resident gut microbiome [27,28,124,125]. In HIV-infected patients, probiotics may provide a beneficial effect [109,110] by restoring the balance of commensals, pathobionts, and pathogens resident at a mucosal surface, as well as by inducing improvements in the epithelial barrier function, to improve CD4 counts and to impact markers of bacterial translocation, inflammation, and immune activation [126,127,128]

There are evidences that beneficial effects of probiotics are strain-dependent and not all interventions are equally effective. It is likely that some probiotic strains adhere better to the small intestine, while others bind specifically to different parts of the large intestine, as well as different strains adhering differently in healthy or injured mucosa. Strictly-related probiotics have shown different in vitro properties, which may mirror differences in clinical effects. 

Thus, immunomodulatory properties of all probiotic bacteria should be characterized in order to develop clinical applications in different target populations [120,121,129]. The recent expansion in the sale and use of probiotics has resulted in an increase in the standards required to scientifically substantiate their claimed beneficial effects. 

Many studies reported that probiotics were “well-tolerated” without side effects, or no statistically significantly increased relative risk of overall number of adverse events. In conclusion: “Across studies, there was no indication that critically ill and high-risk participants taking probiotics were more likely to experience adverse events than control participants with the same health status” [130].

However, additional investigations may provide a full clarification of the mechanism of action by which probiotics can be used as innovative tools to alleviate intestinal inflammation, normalize gut mucosal dysfunction, and downregulate hypersensitivity reactions, with the aim of improving the quality of life during HIV infection, and underlining the economic advantages of probiotic diet supplementation.
